# Poor dietary diversity and low adequacy of micronutrient intakes among rural Indonesian lactating women from Sumedang district, West Java

**DOI:** 10.1371/journal.pone.0219675

**Published:** 2019-07-11

**Authors:** Sofa Rahmannia, Aly Diana, Dimas Erlangga Luftimas, Dida Akhmad Gurnida, Dewi Marhaeni Diah Herawati, Lisa Anne Houghton, Rosalind Susan Gibson

**Affiliations:** 1 Graduate School of Biomedical Sciences Master Program, Faculty of Medicine, Universitas Padjadjaran, Bandung, West Java, Indonesia; 2 Faculty of Medicine, Universitas Pasundan, Bandung, Indonesia; 3 Faculty of Medicine, Universitas Padjadjaran, Bandung, Indonesia; 4 Department of Human Nutrition, University of Otago, Dunedin, New Zealand; CSIR-Foood Research Institute, GHANA

## Abstract

Information on micronutrient adequacy of diets of rural Indonesian lactating women is lacking, despite their high nutrient requirements. This is of concern because deficits in micronutrient intakes may compromise the health of both mothers and infants. This study aimed to assess micronutrient adequacy and dietary diversity (DD) among rural lactating women and explore relationships between micronutrient adequacy, DD, and intakes of energy and food groups consumed. We measured in-home 12-h weighed food records and 12-h recalls over three non-consecutive days from 121 exclusively breastfeeding women at 2–5 months postpartum. Next, we calculated intakes of energy and 11 micronutrients and estimated probability of adequacy (PA) for usual intakes of 11 micronutrients for each women taking into account national fortification of wheat flour with thiamin, riboflavin, folate, zinc, and iron. We assessed DD from nine food groups consumed. Energy and macronutrient balance were within recommended ranges, yet population prevalence of adequacy was less than 50% for niacin, vitamins B6 and C, and less than 60% for calcium, vitamin B12 and vitamin A, all micronutrients not targeted by the national wheat flour fortification program. In contrast, population prevalence of adequacy for the fortified micronutrients was at least 60%, with iron and zinc attaining 79% and 97%, respectively. Overall mean population prevalence of micronutrient adequacy was 57% and mean (±SD) DD score was 4.3±1.2. Mean PAs, a composite measure based on individual PAs over 11 micronutrients, were strongly correlated with energy intakes and with DD scores. In the multivariate models with maternal education and wealth index as covariates, organ meats were the most important determinant of mean PA after controlling for energy intake. In conclusion, despite wheat flour fortification, lactating mothers remained at risk of multiple micronutrient inadequacies. Increasing intakes of animal source foods including organ meats, and fruits and vegetables should be considered.

## Introduction

Breastfeeding imposes additional nutrient demands on lactating mothers to cover both the energy cost of milk production and the nutrients secreted in breast milk [1,2]. As a consequence, the requirements for some micronutrients are increased by 50% or more during lactation [3], making them even higher than for pregnancy [4]. Notwithstanding such high nutrient requirements, information on micronutrient intakes during lactation is limited, especially from low income countries where women are especially at risk to inadequate intakes because their habitual diets are often plant-based and of poor dietary quality [5–9].

Such dietary inadequacies in the face of the augmented micronutrient demands of lactation may compromise the health of both the mothers and their infants. Maternal micronutrient body stores may be depleted leading to co-existing micronutrient deficiencies and adverse health consequences among the mothers [10]. Anemia and biochemical deficiencies of iron, zinc, and vitamin A have been reported in lactating mothers in Asia [7,11] including Indonesia [12,13]. In addition, low maternal intakes have the potential to reduce concentrations of certain micronutrients in breast milk posing risk of suboptimal micronutrient intakes and status among exclusively breastfed infants [12,14].

In 2003, the Indonesian Government instigated mandatory fortification of wheat flour with five micronutrients, namely thiamin, riboflavin, folic acid, iron, and zinc in an effort to overcome dietary deficits [15]. However, to date there is limited information on the adequacy of micronutrients in the diets of rural women in Indonesia who are exclusively breastfeeding or on the factors that may account for any shortfalls in their diets. Previously, we reported on the intakes of energy and micronutrients and their association with breast milk concentrations in a sample of lactating rural Indonesian women [16]. Here, we extend the research by: 1) assessing the adequacy of energy and nutrient intakes in the diets of these same women from rural Indonesia who claimed to be exclusively breastfeeding; 2) characterizing dietary diversity (DD) and their major food sources of energy and micronutrients; and 3) exploring associations between micronutrient intake adequacy, DD, and intake of energy and nine food groups in this population group.

## Methods

### Study design and participants

The participants of this cross-sectional study were lactating women 2–5 months postpartum who claimed to be practicing exclusive breastfeeding at the time of the survey, and who were participating in a study to measure breast milk volume assessed via dose-to-mother deuterium method [17]. The lactating women resided in Tanjungsari, Sumedang district, West Java, Indonesia; details of the study setting have been described earlier [18].

The study was conducted from February to August 2016. Exclusion criteria were women with known chronic illnesses, such as malnutrition, anemia, and tuberculosis. The study sample size was based on the assumption that the prevalence of inadequate intakes of dietary calcium was 75% [13], with a 9% precision, yielding 118 women, selected by convenience sampling. We chose the prevalence of inadequate intakes of calcium because of the very low calcium intakes among lactating women highlighted by earlier research in Indonesia [13] and elsewhere in Asia [5,9] and the absence of calcium as a fortificant in the mandatory fortification of wheat flour in Indonesia. Ethical approval for the study was obtained from the Human Ethics Committees of the Universitas Padjadjaran, Bandung, Indonesia and the University of Otago, Dunedin, New Zealand. Informed written consent was obtained from the participants.

### Socio-demographic characteristic of the mothers and households

Socio-demographic data were obtained through interviews at the community health center by trained research assistants using pre-tested structured questionnaires [19]. Body weight using an electronic scale (Tanita SC-240MA, Maeno-cho, Itabashi-ku, Tokyo, Japan) and height using a stadiometer (SECA 213, Seca GmbH & Co. KG., Hamburg, Germany) were measured using standardized procedures [20], and body mass index (BMI) calculated.

### Dietary data collection

Dietary data were collected over a six-month period that covered both seasonal differences and cultural events in Indonesia. Women were instructed not to change their food consumption patterns during the dietary data collection. Dietary intake data were recorded on three non-consecutive days by trained community health workers (cadres) who conducted 12-hour in-home weighed food records (6 am-6 pm) using calibrated digital scales accurate to ±1 g (Kitchen Scale EK3131, Camry Electronic Ltd, Guangdong, China) combined with maternal 12-hour recall of any foods consumed over the previous 6 pm-6 am period, as described earlier [16] (a detailed protocol has been described elsewhere, see http://dx.doi.org/10.17504/protocols.io.xitfken). The next morning, cadres weighed replicas of the amount of recalled foods or beverages consumed using leftover foods or beverages, where possible. For the small number of irregularly shaped food items with no leftovers, playdough equivalent to the amount of food consumed and of known density, was weighed, allowing the volume of play dough to be estimated. As the volume of the play dough is assumed to be the same as the volume of food eaten, the latter was converted to weight of the actual food consumed using food density data compiled by the investigator or derived from the FAO/INFOODS Density database [21].

### Compilation of a local food composition table

A local Indonesian food composition table (FCT) was compiled for 738 foods, beverages, and mixed dishes using methods described earlier [19]. Micronutrients of interest were vitamin A, thiamin, riboflavin, niacin, vitamin B6, B12, C, folate, calcium, iron and zinc. These micronutrients were selected based on their potential to reduce breast milk concentrations with low maternal intakes [22–26] or the possible consequences arising from deficiencies for the health of both mothers and their infants [10,24,27], as well as evidence from previous reports elsewhere of deficiencies in Asian mothers or their infants [5,6,8,27]. Where appropriate analyzed values for iron, zinc, and calcium based on representative Indonesian staple foods collected from markets and households were included [28]. All micronutrient values for wheat flour products in the FCT were adjusted to take into account the mandatory policy of the Indonesian government to fortify wheat flour with thiamin (2.5 mg/kg), riboflavin (4 mg/kg), iron (50 mg/kg), zinc (30 mg/kg), and folic acid (2 mg/kg) [15], as these adjustments had not been made previously for the food composition data used. Values in the FCT for vitamin A were recorded as retinol activity equivalents (RAE), niacin as mg, and folate as dietary folate equivalents (DFE) to take into account the higher bioavailability of the synthetic folic acid fortificant compared to that for food folate. The average nutrient composition of each mixed dish was calculated based on five recipes per mixed dish [29] collected by the cadres, after adjusting for changes in nutrient retention [30] and yield [31] after cooking, where necessary.

### Assessment of energy and nutrient intakes and probability of adequacy of usual intakes of 11 micronutrients

Median (IQR) daily intakes of energy, macro- and micronutrients were calculated based on the intake data that included the contribution of the five micronutrients from the fortified wheat flour. The median energy intake was compared to the FAO/WHO energy requirement calculated to take into account the basal energy requirement for non-lactating women based on light physical activity and the mean body weight of the lactating women plus the additional demands arising from the production of breast milk [1]. The additional demands were based on the energy cost of milk production assuming an average milk volume of 780 mL/day [32]) adjusted for metabolizable energy. Intakes of protein, fat, and carbohydrate expressed as a percentage of energy were compared with the WHO/FAO [33] acceptable macronutrient distribution ranges.

The Multiple Source method (MSM) program [34] was applied to estimate individual usual intakes of energy and nutrients as well as the usual intake distributions for the study population; for statistical details see Harttig et al. [34]. Next, except for calcium, the probability of adequacy (PA) associated with “usual intake” for each micronutrient and each individual was estimated using the Estimated Average Requirement (EAR) cut-point method. The EARs compiled by WHO/FAO as identified by Arimond et al. [34] were used except for calcium, iron, and zinc. For calcium we chose to estimate PA following the approach of Foote et al. [35] using the U.S Adequate Intake (i.e., 1000 mg/d) [34]. Using this method, the probability of adequacy was defined as follows: 0% for calcium intakes ≤ one fourth of the AI, 25% for calcium intakes > one forth AI and ≤ one half AI, 50% for calcium intakes > one half AI and ≤ three fourths AI, 75% for calcium intakes > three fourths AI and ≤AI, and 100% for calcium intakes above the AI. For zinc, the International Zinc Nutrition Consultative Group (IZiNCG) EAR for lactating women based on a mixed or refined vegetarian diet, assuming 34% bioavailability was used [36]. For iron, the EAR value from the IOM [4,32] adapted assuming 10% bioavailability for these predominantly mixed rice-based diets was used because these exclusively breastfeeding women were assumed not to be menstruating [37].

### Dietary diversity scores and percentage contribution of nine food groups to energy and micronutrient intakes

Dietary diversity (DD) scores were calculated by summing the number of nine food groups consumed on each day by each individual and averaging the three days. A 15-gram intake restriction for inclusion in a food group was applied. Nine food groups were defined: 1) starchy staples; 2) legumes and nuts; 3) dairy products; 4) organ meats; 5) eggs; 6) flesh foods and miscellaneous small animal protein; 7) vitamin-A rich dark green leafy vegetables; 8) other vitamin A-rich fruits and vegetables; 9) all other fruits and vegetables. All foods containing ≥ 60 retinol activity equivalents (RAE)/100g were defined as vitamin A-rich foods. Ingredients in mixed dishes were assigned appropriate food group codes. Food groups that were energy-dense but of a low micronutrient density (i.e., fats and oils; sweets, including sugar drinks) were excluded [37]. Quantitative intakes of the nine food groups (as g/day) and the percentage contribution of each food group to the total energy and micronutrient intakes were calculated and compared.

### Statistical analyses

Descriptive data were presented as percentages or as means and standard deviations except for distributions that were not normally distributed when medians (IQR) were used. Data on maternal education was categorized as primary school or less, secondary school, and college/university. An asset-based wealth index was calculated using principal component analyses as described earlier [18]. A composite measure of micronutrient adequacy for each women termed the mean probability of adequacy (MPA) was calculated by averaging for each women the probability of adequacy estimates across the 11 micronutrients [5]. A population prevalence of adequacy for each micronutrient (expressed as a percentage) was also derived by averaging for each women the estimated probabilities for each of the 11 micronutrients separately. Finally, an overall mean population prevalence of micronutrient adequacy (expressed as a percentage) was calculated by averaging the probabilities of adequacy of the 11 micronutrients from all the individuals.

Associations between MPAs and DD Scores were examined without and with controlling for “usual energy intakes” using Spearman rank correlation coefficients and partial correlation analysis, respectively. To explore which of the nine food groups were the most important determinants of MPA, multivariate quantile regression analysis was conducted to account for any skewed data with and without controlling for energy intake. Maternal education and wealth scores were included as covariates in the models. Data were analyzed using STATA version 12 (Stata Corp, College Station, TX, ESA). Differences were considered significant at P<0.05.

## Results

### Socio-demographic status of the mothers

Descriptive characteristics of the women are shown in Table 1. Of the 121 lactating women, most (81%) were from 2 to 4 months postpartum with the remainder 4 to 5 months postpartum. The mean age (±SD) of the infants was 3.3±0.8 months.

**Table 1:** Descriptive characteristics of lactating women.

Descriptive characteristics	n	%
**Age, y** (Median, IQR)	121	25 (21–30)
**Post-partum, months** (Median, IQR)	121	3.3 (2.6–3.7)
**Maternal education level**		
No schooling	2	1.7
Primary	45	37.2
Secondary	67	55.4
College/University	7	5.8
**Spouse education level**		
No schooling	1	0.8
Primary	53	43.8
Secondary	57	47.1
College/University	10	8.3
**Maternal occupation**		
Housewife	103	85.1
Others	18	14.9
**Spouse occupation**		
Regular wage earner	30	24.8
Business or trader	23	19.0
Manual worker	16	13.2
Farmer	22	18.2
Unemployed	30	24.8
**Number of household members**		
3–4	73	60.3
5–6	36	29.8
≥7	12	9.9
**BMI**^a^		
Underweight	7	5.8
Normal	72	59.5
Overweight/obese	42	34.7

n: Number of participants

^a^WHO Expert Consultation, 2004

### Intakes of energy, macronutrients and prevalence of micronutrient adequacy

The reported median intake of energy (i.e., 2165 kcal) was very close to the recommended average requirement for exclusively breastfeeding women assuming light physical activity, calculated according to FAO/WHO [1]. The contribution of protein, fat, and carbohydrate as a percentage of energy was also within the WHO/FAO [33] recommended ranges (Table 2).

**Table 2:** Energy and macronutrient intakes compared with recommendations.

	Energy (kcal)	Protein (g)	Fat (g)	Carbohydrate(g)
Mean (Standard deviation)	2211 (578)	73.6 (21.0)	57.8 (17.7)	345 (98)
Median (range)	2165 (1004–3496)	70.7 (33.1–140.7)	56.6 (19.4–128.3)	338 (130–589)
Macronutrient as percent energy intake	N/A	13%	24%	62%
WHO/FAO recommendation^a^	2472	10–15%	15–30%	55–75%

^a^WHO/FAO, 2003

The overall mean prevalence of adequacy for the population of women across the 11 micronutrients was 57±28% (Table 3). The prevalence of adequacy for each of the micronutrients not targeted by fortification was less than 50% for niacin, vitamin B6, and vitamin C, and less than 60% for calcium, vitamin B-12, and vitamin A. The prevalence of adequacy for each of the micronutrients targeted by fortification was at least 60%, with iron and zinc attaining 79% and 97%, respectively.

**Table 3:** Intakes and population prevalence of adequacy of micronutrients (as %).

Nutrient	EAR	Median	IQR	Prevalence of adequacy, %
Vitamin A (RAE)	450	501	(319–841)	57
**Thiamin (mg)**^a^	**1.2**	**1.4**	**(0.98–1.84)**	**60**
**Riboflavin (mg)**^a^	**1.3**	**1.7**	**(1.2–2.2)**	**69**
Niacin (mg)^a^^b^	13	12.8	(10.1–15.5)	47
Vitamin B6 (mg)^a^	1.7	1.3	(1.0–1.7)	25
**Folate (μg)**	**450**	**618**	**(478–836)**	**79**
Vitamin B12 (μg)	2.4	2.5	(1.8–3.1)	52
Vitamin C (mg)	58	38	(25–62)	28
Calcium (mg)^c^	-	613	(509–750)	51
**Iron (mg)**^d^	**11.7**	**18.3**	**(12.5–23.0)**	**79**
**Zinc (mg)**^e^	**7**	**12.8**	**(10.8–15.0)**	**97**
Overall mean population prevalence of adequacy^f^				57
Standard deviation				28

**Bold** indicates micronutrients targeted by fortification of wheat flour

EAR: Estimated average requirement

All values for EARs are from WHO/FAO (2004) unless otherwise stated.

^a^Back calculated from Recommended Nutrient Intake (RNI) (WHO/FAO, 2004)

^b^Does not include niacin from tryptophan

^c^ Based on adequacy of intake assessed using the method of Foote et al. [35]

^d^EAR from IOM (2001) assuming 10% bioavailability

^e^EAR from IZiNCG (2004) assuming bioavailability from a mixed or refined vegetarian diet

^f^ Overall mean population prevalence of adequacy is the mean of the adequacies of the 11 micronutrients for all women

### Dietary diversity and major food sources of energy and micronutrients

The Mean Dietary Diversity Score (MDDS) over 3 days was 4.3±1.2 food groups. The food groups most commonly consumed on average were starchy staples (mainly rice), followed by flesh foods/other meats and legumes and nuts. The percentage of women consuming organ meats and dairy products was low, ranging from 7 to 20%, respectively (Table 4). Starchy staples provided the major source of energy (68%) and most micronutrients, except for vitamins B12, A, and C (Table 5).

**Table 4:** Mean dietary diversity score, percentage of women consuming, and weight of each of the nine food groups consumed averaged over 3 days.

Lactating women (n = 121)		
Mean	4.3	
Standard deviation	1.2	
Median	4.3	
Minimum	1.7	
Maximum	6.7	
Consumption	%	Median (IQR), gram
Starchy staples	100	903 (726–1097)
Legumes and nuts	69	86 (37–130)
Dairy products	20	0 (0–64)
Organ meats	7	0 (0–0)
Eggs	53	36 (16–59)
Other meats/flesh foods	78	87 (51–139)
Vitamin A-rich leafy green vegetables	14	0 (0–14)
Vitamin A-rich fruits and vegetables	40	56 (0–126)
Other fruits and vegetables	55	51 (17–129)

**Table 5:** Percentage of energy and micronutrients from food sources.

Food groups	Energy	Calcium	Vit.A	Niacin	Vit.B6	Vit.B12	Vit.C	Iron	Zinc	Thiamin	Riboflavin	Folate
Starchy staples	68	31	8	46	41	6	14	52	62	65	50	76
Legumes and nuts	7	28	1	7	9	1	4	15	8	6	8	7
Dairy products	2	9	2	0	1	4	1	7	1	2	9	0
Organ meats	0	0	19	5	2	23	1	2	2	1	4	2
Eggs	5	5	8	1	5	14	0	5	4	6	11	4
Other meats/flesh foods	10	14	5	33	25	50	3	12	17	15	11	4
Vitamin A-rich leafy green vegetables	0	2	4	1	1	0	4	1	0	0	1	1
Vitamin A-rich fruits and vegetables	3	6	52	4	7	1	51	4	2	3	3	3
Other fruits and vegetables	2	3	1	3	8	0	21	1	1	2	3	2
Others	3	2	1	1	1	1	1	1	1	0	1	0

### Associations between DD scores, energy intake, food groups and mean probability of adequacy

The MPA for the individuals were highly correlated with energy intakes (r = 0.791, P<0.001) and with DD scores(r = 0.723, P = <0.001), although the latter relationship was weakly attenuated after controlling for usual energy intake (i.e., r = 0.564, P<0.001). In the multivariate models with maternal education and wealth index as covariates, organ meats were the most important determinant of MPA, after controlling for usual energy intake. For each unit increase (100g) in maternal intake of organ meats, MPA increased by 37% (Table 6).

**Table 6:** Associations of mean probability of adequacy of micronutrient intakes with individual food groups.

Variable	Mean difference in MPA per unit increase (95% CI)	P-value
Starchy staples*	-0.5 (-2.8, 1.9)	0.708
Legumes and nuts*	2.5 (-2.8, 7.8)	0.349
Dairy products*	1.5 (-3.6, 6.6)	0.553
Organ meats*	36.8 (2.6, 71.0)	**0.035**
Eggs*	2.6 (-8.8, 14.1)	0.648
Other meats/flesh foods*	1.9 (-3.7, 7.5)	0.508
Vitamin A-rich leafy green vegetables*	-0.7 (-15.4, 14.0)	0.926
Vitamin A-rich fruits and vegetables*	5.2 (1.2, 9.3)	**0.012**
Other fruits and vegetables*	5.9 (0.8, 11.0)	**0.023**
Energy intake*	2.6 (1.0, 4.2)	**0.002**
Wealth score	-0.8 (-3.2, 1.6)	0.493
Maternal education	6.7 (0.6, 12.7)	**0.030**

Note:

*per 100 gram increase

MPA: mean probability of adequacy for each individual based on 11 micronutrients, expressed as a percentage

**Bold** indicates P<0.05

## Discussion

Of the 11 micronutrients, the mean prevalence of adequacy among these lactating women was less than 60% for six micronutrients, most notably for vitamins B6 and C, followed by niacin, calcium, B12, and vitamin A, despite an average energy intake and macronutrient balance that appeared acceptable [33]. In contrast, for the remaining five micronutrients for which intakes were augmented by consumption of fortified wheat flour, albeit only 70 grams/capita/day, the prevalence of adequacy was at least 60%, with more than 79% of the women having adequate intakes of folate and iron, and 97% for zinc. As a consequence, the overall population prevalence of micronutrient adequacy for the lactating women studied here was markedly higher and their diet more diverse compared to reports in several other low income countries [5,9,38,39]. Moreover, the MPAs among the women were strongly associated with dietary diversity, with organ meats being the strongest predictor of MPA after controlling for usual energy intake.

The low prevalence of adequate intakes of vitamin C intakes noted here is consistent with findings in other Asian countries [5,37,39], and linked to the low intakes of fresh fruits and vegetables. Such low intakes of vitamin C have the potential to compromise absorption of non-heme iron, the major source of iron in these Indonesian diets (Table 5); only 12 to 14% of dietary iron was supplied by flesh foods, a source of readily available heme iron [40]. The consumption of flesh foods was also relatively low and contributed to the low adequacy of intakes of vitamins B6, B12, and niacin by these lactating women [40] (Table 4). Such low maternal intakes of B6, B12, and niacin during lactation have the potential to reduce their secretion in breast milk [22] posing risk of inadequacy for these exclusively breastfed infants [25,41,42].

Only 51% of these Indonesian lactating women had adequate calcium intakes, although even lower estimates have been reported in earlier studies [13,37,38], some of which were based on the approach used here [37]. However, in many of these earlier studies [5,13,37–39] both the frequency and average amount of dairy products consumed were even lower than reported here. Hence, the suggestion that dietary calcium may influence breast milk calcium concentrations [43] warrants further investigation especially among lactating women in Asia whose calcium intakes are habitually low.

The adequacy of vitamin A intakes, although only 57%, was also greater here than elsewhere in Asia [5,37–39], even though none of the women had received high-dose vitamin A supplements postpartum in line with the recent WHO recommendation [44] that encourages lactating women to only consume a healthy balanced diet. Deep orange/yellow fruits and vegetables provided 50% of the vitamin A, the remainder being contributed from organ meats and eggs; the quantity of dark green leafy vegetables consumed was very low.

Very few women had inadequate intakes of zinc, a finding consistent with the low risk of suboptimal biochemical zinc status reported earlier among lactating women in Indonesia [13] and among our Sumedang infant cohort [18] compared to that in other Asian countries [11,39,45,46]. Consumption of large quantities of rice grown in Sumedang district where soil zinc concentrations are high [47] in addition to wheat flour fortified with zinc were probably contributing factors. In contrast to zinc, although nearly 80% of the women seemingly had adequate intakes of iron, there are reports of sub-optimal iron status among lactating mothers [13] and their infants in Indonesia [18]. Several factors may be responsible for poor iron status in the face of seemingly adequate iron intakes, including poor bioavailability of iron from both plant-based foods and the elemental iron fortificant used to fortify wheat flour [48]. Poor absorption of dietary iron arising from inflammation induced by infection [18] and maternal overweight/obesity may be additional contributing factors [49]. The prevalence of adequate intakes of folate was also, like iron, almost 80%, contributed in part by the folic acid added to wheat flour (200 ug/100 g) as a fortificant in view of the well-established association between inadequate folate intake and neural tube defects.

### Dietary diversity and associations with overall micronutrient intake adequacy

Earlier reports from Bangladesh [39] and the Philippines [37] reported weaker correlations between dietary diversity and MPAs compared to the diets of these Indonesian lactating women, with vitamin A-rich fruits and vegetables being the strongest predictor of MPA among Bangladeshi women [39] in contrast to organ meats noted here.

### Strengths and limitations

We believe our study has several strengths. Our dietary data are based on three non-consecutive days, and our probability of adequacy estimates for the 11 micronutrients were calculated directly from the distribution of estimated individual usual intakes. Nevertheless, the adequacy of niacin intakes is underestimated here because the contribution of niacin from tryptophan was not included due to limitations in the Indonesian food composition data [40]. In addition, as noted earlier, our study is also limited by the absence of data on the micronutrient status of the lactating women. We also recognize that our participants were not based on a representative sample of lactating women in Sumedang district, and our cross-sectional study design prevents causal inferences from being made.

In conclusion, this study highlights that these lactating Indonesian mothers were at risk of multiple micronutrient inadequacies, only some of which were targeted by the national wheat flour fortification program. Strategies to increase the consumption of animal-source foods including organ meats, as well as fresh fruits and vegetables should be considered to reduce the shortfalls in niacin, vitamins B6, B12, A, C and calcium identified in the diets of these lactating women.

## Supporting information

S1 Dataset (RAR)
